# Assessment the Predictive Value of Left Atrial Strain (LAS) on Exercise Tolerance in HCM Patients with E/e' between 8 and 14 by Two-Dimensional Speckle Tracking and Treadmill Stress Echocardiography

**DOI:** 10.31083/j.rcm2406167

**Published:** 2023-06-08

**Authors:** Ye Su, Chunmei Li, Lixue Yin

**Affiliations:** ^1^School of Medicine, University of Electronic Science and Technology of China, 611730 Chengdu, Sichuan, China; ^2^Department of Cardiovascular Ultrasound, Sichuan Provincial People’s Hospital, University of Electronic Science and Technology of China, 610031 Chengdu, Sichuan, China; ^3^Ultrasound in Cardiac Electrophysiology and Biomechanics Key Laboratory of Sichuan Province, Sichuan Provincial People’s Hospital, University of Electronic Science and Technology of China, 610031 Chengdu, Sichuan, China

**Keywords:** HCM, reservoir strain, conduit strain, contraction strain, metabolic equivalent

## Abstract

**Background:**

The aim of this study was to evaluate the reservoir, conduit, and contraction function of the left atrium and to evaluate the predictive value of left atrial strain (LAS) on exercise tolerance in hypertrophic cardiomyopathy (HCM) patients with an E/e’ between 8 and 14 by two-dimensional speckle tracking using treadmill stress echocardiography.

**Methods:**

This was a retrospective study in which we analyzed a total of 70 patients with HCM between 2016 and 2017. According to the resting state E/e’, patients were either assigned to an HCM-1 group (E/e’ >14) or an HCM-2 group (E/e’ of 8 to 14). Thirty age-matched healthy controls were included in the normal group. Analysis involved the left atrial reservoir, conduit, contraction strain and reserve function.

**Results:**

The normal group had a higher left atrial reservoir and conduit strain than the HCM-2 group; the lowest values were in the HCM-1 group. The LAS reserve capacity of the HCM-1 and HCM-2 groups was lower than those of the normal group. The left atrial contraction strain reserve (ΔLASct%) and global longitudinal strain reserve (ΔGLS%) were lower in the HCM-2 and HCM-1 groups than in the normal group. We also found that the ΔLASct% and ΔGLS% in the HCM-2 group were higher than in the HCM-1 group. Furthermore, the metabolic equivalents (METS) in the HCM-2 group was greater than that in the HCM-1 group. Finally, the Rest-LASr indicated the highest differential diagnostic performance for METS <6.0 (area under curve [AUC]: 0.759); the AUC of the composite model Rest-LASr+E/e’-rest was 0.8.

**Conclusions:**

Analysis showed that when the E/e’ was between 8 and 14, the LAS and reserve capacity of HCM patients were significantly reduced. Our findings suggest that the routine assessment of LAS +E/e’ can be a strategy with which to supplement current predictive models and facilitate clinical management strategies.

## 1. Introduction

Hypertrophic cardiomyopathy (HCM) is a hereditary form of cardiomyopathy. Patients with HCM often have a range of clinical symptoms, including palpitations, dyspnea, and reduced exercise tolerance. An abnormal left ventricular (LV) diastolic function is an early pathophysiological change and an important cause of HCM progression. At present, echocardiography is the preferred non-invasive imaging method for the evaluation of LV diastolic function; the core indicator is E/e’. The American Society of Echocardiography/European Society 2016 Guidelines for Cardiovascular Imaging recommended an E/e’ >14 as the cut-off value for increased left ventricular filling pressure and an E/e’ <8 as the normal cut-off value [1]. Previous studies on LV diastolic function in HCM mainly focused on its causes and its impact on prognosis, while less attention was paid to exercise capacity and the tolerance of patients with HCM and an E/e’ of 8–14. Left atrial function plays an important role in the filling pressure of the left ventricle. Two dimensional Speckle tracking Technology (2DSTI) can sensitively and specifically evaluate LA function by analyzing LA strain (LAS). Over recent years, LAS has been shown to have the potential to independently assess LV diastolic function in a rapid and simple fashion [2]. However, LA function is very sensitive to stress [3]. Therefore, in this study, we used treadmill stress echocardiography combined with 2DSTI to evaluate the LA reservoir, conduits, contraction function, and its impact on metabolic equivalents (METS) in HCM patients with an E/e’ ranging from 8 to 14.

## 2. Materials and Methods

### 2.1 Research Subjects

**Fig. 1.**
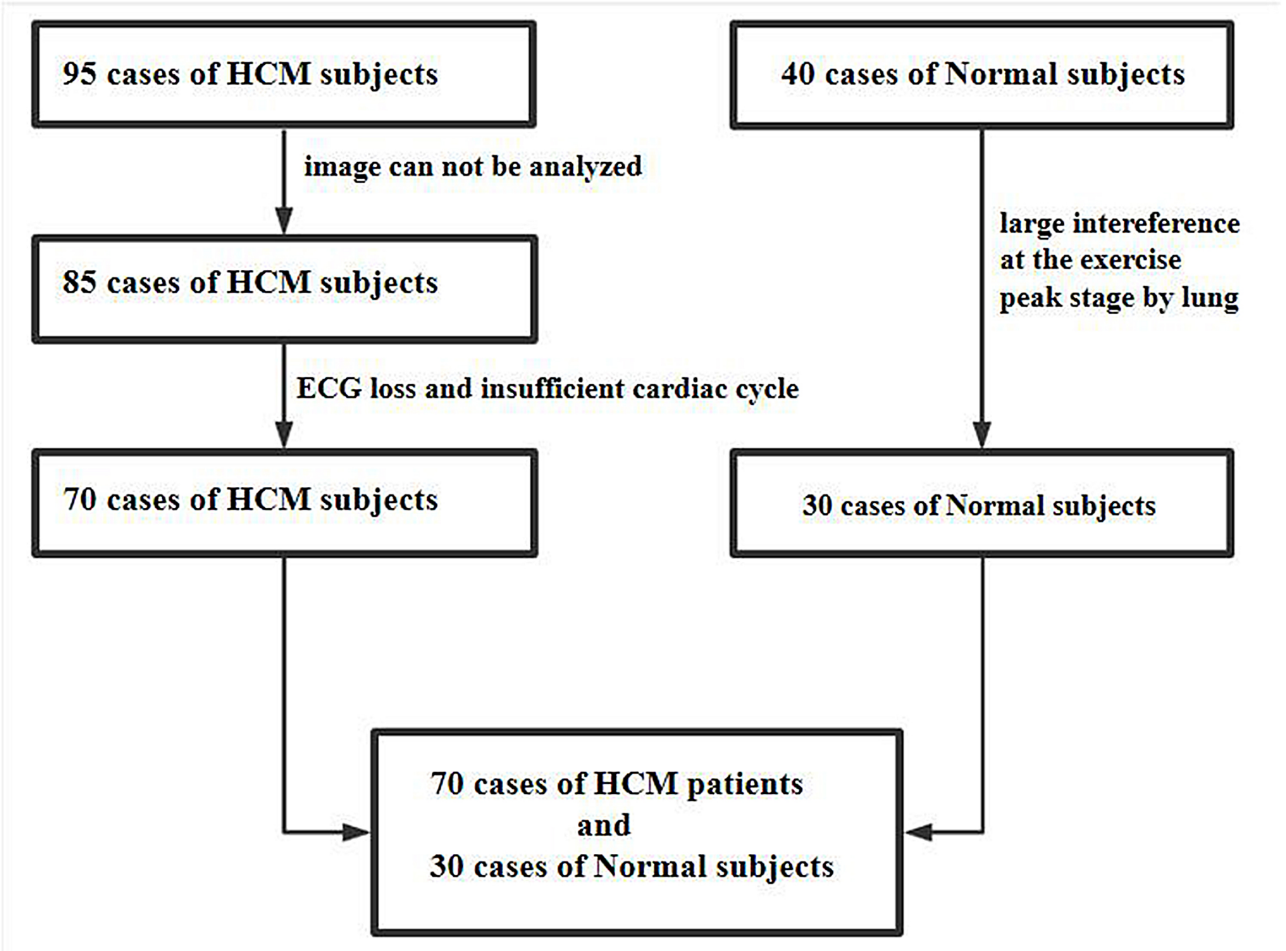
**A flow chart showing patient recruitment**. A total of 95 patients with HCM were initially included in the study, 10 patients were excluded because the peak image could not be analyzed, 15 patients were excluded because of insufficient cardiac cycle of image and ECG loss, finally, 70 patients with HCM were included in the study. In the normal group, 40 cases were initially included, 10 cases were excluded due to the large interference at the peak stage by the lung, finally, 30 cases were included with the image quality meeting the requirements. HCM, hypertrophic cardiomyopathy; ECG, electrocardiogram.

This was a retrospective analysis of a total of 70 HCM patients who underwent treadmill exercise stress ultrasound evaluation in Sichuan Provincial People’s Hospital from 2016 to 2017 and included 45 males and 25 females, with a mean age of 47 ± 15 years. The inclusion criteria were as follows: HCM diagnosed according to the 2017 Guidelines for the Diagnosis and Treatment of Hypertrophic Cardiomyopathy in Chinese Adults and the 2014 ESC Guidelines, with a resting E/e’ value >7 by conventional echocardiography. The exclusion criteria were as follows: patients with hypertension, severe arrhythmia, ischemic heart disease, congenital heart disease, respiratory system disease and other diseases that affect cardiac function; other contraindications related to treadmill stress echocardiography [4], and patients with poor imaging quality. According to a resting state E/e’ >14, the patients were assigned to the HCM-1 group, while those with an E/e’ of 8–14 were assigned to the HCM-2 group. In addition, we included 30 normal controls who underwent treadmill exercise stress echocardiography, including 15 males and 14 females, with a mean age of 46 ± 11 years (See Fig. 1 for a flow chart showing patient recruitment). This study was approved by the ethics committee of our hospital, and all patients signed the informed consent for treadmill exercise stress test.

### 2.2 Electrocardiography of Treadmill Exercise

Symptom-restricted exercise tests were performed by SunTechTango synchronized ambulatory hemometry (SunTech Medical Instruments, NC, USA) and a MortaraX-Scribe treadmill exercise analysis system (Mortara Instrument, Milwaukee, WI, USA) using the BRUCE protocol. Electrocardiograms (ECGs) and blood pressure were monitored during exercise. All subjects were asked to stop β-blockers or calcium channel blockers for at least 24 hours before the trial. Resting contraction and diastolic blood pressure were measured, and ECGs were recorded simultaneously. Exercise termination metrics were based on the 2002 ACC/AHA Exercise Testing Guidelines Update [5]. Previous studies [6, 7] reported that an estimated metabolic equivalent (MET) <6.0 represented the cut-off value to evaluate impaired exercise tolerance and had the highest predictive value for all-cause mortality. In this study, HCM patients were divided into two categories with a METS >6.0 and <6.0 for further analysis.

### 2.3 Exercise Stress Echocardiography

For exercise stress electrocardiography, we used a Philips EPIQ7C ultrasonic diagnostic apparatus and X5-1 probe (1.0~5.0 MHz) full-function pure wave single crystal matrix probe; apical four-chamber, three-chamber, and two-chamber dynamic images from at least five cycles at rest and peak state were collected. All parameters were measured and analyzed in accordance with American Society of Echocardiography (ASE) guidelines [8, 9, 10]; body surface area (BSA), body mass index (BMI), left ventricular end-diastolic volume (EDV), left ventricular end-systolic volume (ESV), left ventricular ejection fraction (EF), ejection fraction reserve (ΔEF% = (Peak_EF-Rest_EF)/Rest_EF), left ventricular interventricular septum thickness (IVS), left ventricular posterior wall thickness (LVPW), left atrial diameter (LA), peak early (E), late (A) mitral inflow velocity, E/A ratio, peak early-diastolic mitral annular velocity (e’), E/e’ ratio (e’ was calculated as the mean of the septal e’wave and lateral e’wave by using pulsed wave-tissue Doppler imaging) were determined as indices for LV filling pressures. Offline software QLAB13 (Philips Netherlands) was used for strain analysis. LA strain analysis utilized “AutoStrain LA” in QLAB13 offline software, which is based on the two-dimensional (2D) speckle tracking technology. The first step involved selecting the clear apical four-chamber dynamic image and importing this to “AutoStrain LA”. The second step confirmed the inner boundary of the left atrium and made manual adjustments according to the dynamic image obtained in the first step. The final step involved an auto calculation process to determine an accurate strain value. The QRS complex (R-R gating) was used to initiate the strain calculation. When the R-R gating was used, the LASr values were positive, the LAScd and LASct values were negative. The difference between reservoir strain and atrial contractile strain values is known to reflect conduit function [11]. According to standardization of left atrial, a consensus document of the EACVI/ASE/Industry Task Force to standardize deformation imaging [12]: LA reservoir strain (LASr), LA conduit strain (LAScd), LA contraction strain (LASct), at the same time, the LA reservoir strain reserve (ΔLASr% = (Peak_LASr-Rest_LASr)/Rest_LASr), LA conduit strain reserve (ΔLAScd% = (Peak_LAScd-Rest_LAScd)/Rest_LAScd), and LA contraction strain reserve (ΔLASct% = (Peak_LASct-Rest_LASct)/Rest_LASct) were calculated. LV strain analysis utilized “AutoStrain LV” in QLAB13 offline software: LV global longitudinal strain (GLS), LV global longitudinal strain reserve (ΔGLS% = (Peak_GLS-Rest_GLS)/Rest_GLS), ΔGLS=Peak_GLS-Rest_GLS, Heart rate (HR), Systolic blood pressure (SBP), Diastolic blood pressure (DBP), left atrial volume index (LAVI).

### 2.4 Statistical Methods

All statistical analyses were performed using SPSS 23.0 software (IBM SPSS Statistics, version 23, Armonk, NY, USA) and R for WINDOWS 4.0.3 software (R Development of Core Team, the terms of the Free Software Foundation’s GNU General Public License). Continuous variables are expressed as mean ± standard deviation. Between-group comparisons were performed by independent samples *t*-test and within-group pre- and post-exercise comparisons were performed by paired *t*-test, and values of *p* < 0.05 were considered statistically significant. Intraclass correlation coefficient (ICC) was used for the intra- and interobserver variability analysis. The sensitivity and specificity of the variables to METS were analyzed after binary logistic regression to determine the effects of variables on exercise tolerance.

## 3. Results

### 3.1 Comparison of Characteristics and Ultrasound Parameters 

The mean age of the 70 HCM patients was 47.14 ± 15.04 years. All HCM patients were in sinus rhythm on ECG. In the HCM-1 group (a total of 36 cases with an E/e’ >14) included 16 cases with tricuspid regurgitation grade I (44.44%), five cases with tricuspid regurgitation grade II (13.89%), 12 cases without tricuspid regurgitation (33.33%), 23 cases with mitral regurgitation grade I (accounting for 63.89%), 10 cases with mitral regurgitation grade II (accounting for 27.78%), 0 cases of grade III mitral regurgitation, five cases (13.89%) with left ventricular outflow tract obstruction (LVOT) at resting level (Vmax >2.74 m/s, PG >30 mmHg), 31 cases with left atrial volume index (LAVI) >34 mL/$m^{2}$ (86.11%), 29 cases of asymmetric hypertrophy (80.55%), and eight cases of apical hypertrophy (22.22%). In the HCM-2 group (a total of 36 cases with an E/e’ ranging from 8 to 14) included 20 cases with tricuspid regurgitation grade I (58.82%), five cases with tricuspid regurgitation grade II (accounting for 14.70%), 12 cases without tricuspid regurgitation (accounting for 35.29%), 30 cases with mitral regurgitation grade I (accounting for 88.23%), five cases with mitral regurgitation grade II (accounting for 14.70%), two cases of mitral regurgitation grade III (5.88%), 17 cases (50%) with LAVI >34 mL/$m^{2}$, 0 cases with left ventricular outflow tract obstruction at resting level, 28 cases of asymmetric hypertrophy (82.35%) and five cases of apical hypertrophy (14.70%). A total of 5 cases of HCM had LVOT at rest; all of these were in the HCM-1 group; 11 cases had LVOT after exercise, eight cases in the HCM-1 group and three cases in the HCM-2 group. As shown in Table 1, LA, LAVI, end-diastolic volume (EDV)-rest and E/e’-rest in the HCM group were significantly higher that in the normal group in the resting state; LVPWT and IVS in the HCM group were significantly thicker than those in the normal group (*p <* 0.05); and ΔEF% in the HCM group was significantly lower than that in the normal group (*p <* 0.05). As shown in Table 2, comparison of conventional echocardiographic parameters between the HCM-1, HCM-2 and normal groups showed that LA and LAVI in the HCM-1 group were significantly higher than those in the normal group (*p <* 0.05) and that LA and LAVI in the HCM-2 group were higher than those in the normal group (*p <* 0.05), but remained within the normal reference range [13]. Previous studies have shown that strain is more sensitive to cardiac function than conventional parameters. Therefore, there is still a need to investigate left atrial and left ventricular strain in patients with HCM.

**Table 1.** **Comparison of general information and echocardiography parameters between HCM group and normal group**.

	HCM group (N = 70)	Normal group (N = 30)	*p*
Age (year)	47.14 ± 15.04	46.60 ± 11.41	0.850
BSA	1.67 ± 0.16	1.68 ± 0.44	0.920
BMI	23.56 ± 3.26	21.96 ± 2.22	0.330
LA (mm)	37.09 ± 5.49	31.47 ± 3.02	0.000*
LAVI (mm/$m^{2}$)	37.2 ± 5.09	23.87 ± 2.64	0.000*
LVPW (mm)	12.83 ± 1.47	8.17 ± 1.08	0.000*
LV (mm)	40.69 ± 7.56	42.57 ± 3.07	0.100*
IVS (mm)	17.21 ± 1.56	9 ± 1.11	0.000*
E-rest (m/s)	0.70 ± 0.18	0.88 ± 0.17	0.000*
A-rest (m/s)	0.73 ± 0.27	0.64 ± 0.12	0.080
E/A-rest (m/s)	1.09 ± 0.49	1.40 ± 0.22	0.010*
e-rest (m/s)	0.05 ± 0.02	0.11 ± 0.01	0.000*
EDV-rest (mL)	91.76 ± 25.29	77.37 ± 19.89	0.010*
ESV-rest (mL)	25.30 ± 10.14	26.47 ± 7.87	0.580
EDV-peak (mL)	88.43 ± 24.52	67.30 ± 18.76	0.000*
ESV-peak (mL)	15.50 ± 10.59	13.97 ± 4.77	0.450
EF-rest (%)	0.72 ± 0.06	0.66 ± 0.04	0.000*
EF-peak (%)	0.83 ± 0.10	0.79 ± 0.05	0.080
ΔEF	0.10 ± 0.05	0.13 ± 0.04	0.070
ΔEF (%)	0.14 ± 0.01	0.20 ± 0.06	0.020*
E/e’-rest	14.36 ± 5.34	6.04 ± 1.08	0.000*
METS	9.05 ± 2.68	10.46 ± 2.13	0.012*
HR-rest	79.23 ± 12.96	71.07 ± 8.48	0.002*
Rest-SBP	126.95 ± 24.20	120.34 ± 8.13	0.041*
Rest-DBP	76.99 ± 12.63	78.04 ± 8.50	0.587
Peak-SBP	172.94 ± 27.84	168.94 ± 14.97	0.323
Peak-DBP	75.59 ± 16.75	77.56 ± 11.42	0.391
Rest-HR	79.23 ± 12.96	71.07 ± 8.48	0.002*
Peak-HR	172.56 ± 15.04	173.40 ± 6.38	0.801

*p < 0.05. EDV-rest, end-diastolic volume at rest stage; ESV-rest, end-systolic volume at rest stage; EDV-peak, end-diastolic volume at peak stage; ESV-peak, end-systolic volume at peak stage; Rest-SBP, systolic blood pressure at rest stage; Rest-DBP, diastolic blood pressure at rest stage; Peak-SBP, systolic blood pressure at peak stage; Peak-DBP, diastolic blood pressure at peak stage; LAVI, left atrial volume index; BSA, body surface area; BMI, body mass index; LA, left atrial diameter; LVPW, left ventricular posterior wall thickness; LV, left ventricular diameter; IVS, left ventricular interventricular septum thickness; E-rest, Early diastolic forward mitral flow velocity at rest stage; A-rest, Late diastolic mitral valve forward flow velocity at rest stage; e’-rest, early-diastolic mitral annular velocity (e’ was calculated as the mean of the septal e’wave and lateral e’wave by using pulsed wave-tissue Doppler imaging) at rest stage; EF, left ventricular ejection fraction; ∆EF = (EF_peak-EF_rest); ∆EF% = (EF_peak-EF_rest)/EF_rest); METS, estimated metabolic equivalent; Rest-HR, heart rate at rest stage; Peak-HR, heart rate at peak stage; HCM, hypertrophic cardiomyopathy.

**Table 2.** **Comparison of general information and echocardiographic parameters among HCM-1, HCM-2, and normal groups**.

	HCM-1 (N = 36)	HCM-2 (N = 34)	Normal (N = 30)	*p* (1-2)	*p* (1-N)	*p* (2-N)
age (year)	50.94 ± 14.68	43.12 ± 14.55	46.60 ± 6.38	0.028*	0.137	0.231
BSA	1.67 ± 0.18	1.66 ± 0.14	1.68 ± 0.04	0.901	0.277	0.291
BMI	24.09 ± 3.14	22.99 ± 3.33	21.96 ± 2.22	0.159	0.158	0.157
LA (mm)	39.56 ± 5.13	34.47 ± 4.62	31.47 ± 3.02	0.000*	0.000*	0.004*
LAVI (mm/$m^{2}$)	39.78 ± 5.24	34.47 ± 3.18	23.87 ± 2.64	0.000*	0.000*	0.000*
LVPW (mm)	12.31 ± 1.94	13.38 ± 1.86	8.17 ± 1.08	0.551	0.000*	0.000*
LV (mm)	40 ± 7.40	41.44 ± 7.76	42.57 ± 3.07	0.441	0.082	0.460
IVS (mm)	18.19 ± 6.79	16.06 ± 6.19	9 ± 1.11	0.187	0.000*	0.000*
E-rest (m/s)	0.72 ± 0.20	0.67 ± 0.15	0.88 ± 0.17	0.295	0.001*	0.000*
A-rest (m/s)	0.82 ± 0.31	0.64 ± 0.19	0.64 ± 0.12	0.008*	0.006*	0.997
E/A-rest	1.02 ± 0.50	1.17 ± 0.48	1.4 ± 0.22	0.207	0.000*	0.018*
E/e’-rest	18.24 ± 4.45	10.25 ± 2.20	6.04 ± 1.08	0.000*	0.000*	0.000*
E/e’-peak	17.43 ± 7.09	12.29 ± 3.86	6.08 ± 0.82	0.000*	0.000*	0.000*
EDV-rest (mL)	93.06 ± 23.45	90.52 ± 27.29	77.36 ± 19.89	0.689	0.006*	0.033*
ESV-rest (mL)	24.70 ± 8.06	25.88 ± 11.95	26.46 ± 7.87	0.635	0.382	0.823
EDV-peak (mL)	90.42 ± 23.63	86.44 ± 25.58	67.30 ± 18.76	0.508	0.000*	0.001*
ESV-peak (mL)	16.38 ± 13.01	14.62 ± 7.54	13.96 ± 4.77	0.493	0.340	0.683
METS	7.91 ± 2.76	10.25 ± 2.00	10.46 ± 2.13	0.000*	0.000*	0.690
HR-rest (bpm)	78.03 ± 12.09	80.50 ± 13.89	71.07 ± 8.48	0.429	0.010*	0.002*
HR-peak (bpm)	169.06 ± 14.68	176.88 ± 14.55	173.40 ± 6.38	0.028*	0.115	0.213

*p < 0.05. EDV-rest, end-diastolic volume at rest stage; ESV-rest, end-systolic volume at rest stage; EDV-peak, end-diastolic volume at peak stage; ESV-peak, end-systolic volume at peak stage; LAVI, left atrial volume index; BSA, body surface area; BMI, body mass index; LA, left atrial diameter; LVPW, left ventricular posterior wall thickness; LV, left ventricular diameter; IVS, left ventricular interventricular septum thickness; E-rest, Early diastolic forward mitral flow velocity at rest stage; A-rest, Late diastolic mitral valve forward flow velocity at rest stage; e’-rest, early-diastolic mitral annular velocity (e’ was calculated as the mean of the septal e’wave and lateral e’wave by using pulsed wave-tissue Doppler imaging) at rest stage; e’-peak, early-diastolic mitral annular velocity (e’ was calculated as the mean of the septal e’wave and lateral e’wave by using pulsed wave-tissue Doppler imaging) at peak stage; METS, estimated metabolic equivalent; HR-rest, heart rate at rest stage; HR-peak, heart rate at peak stage; HCM, hypertrophic cardiomyopathy.

### 3.2 Comparison of Left Atrium and Left Ventricular Strain and Reserve Function between HCM and Normal Group 

As shown in Table 3: Rest-LASr, Rest-LAScd, Rest-LASct, Peak-LASr, Peak-LAScd, Peak-LASct, GLS, ΔGLS, ΔGLS%, ΔLASr%, ΔLAScd%, ΔLASct% in the HCM group were significantly lower than those in the normal group (*p <* 0.05).

**Table 3.** **Comparison of strain between HCM group and normal group**.

	HCM group (N = 70)	Normal group (N = 30)	*p*
Rest-LASr	21.8 ± 6.33	44.39 ± 8.20	0.000*
Rest-LAScd	–13.81 ± 7.52	–27.97 ± 6.45	0.000*
Rest-LASct	–7.71 ± 1.71	–16.41 ± 3.73	0.000*
Peak-LASr	20.28 ± 7.21	66.61 ± 10.90	0.000*
Peak-LAScd	–12.77 ± 8.74	–38.32 ± 7.47	0.000*
Peak-LASct	–6.44 ± 1.41	–28.28 ± 9.20	0.000*
Rest-GLS	–20.07 ± 2.95	–25.25 ± 2.27	0.000*
Peak-GLS	–18.91 ± 6.09	–35.67 ± 2.50	0.000*
ΔGLS	–1.1 ± 0.67	10.4 ± 3.13	0.000*
ΔGLS%	–0.05 ± 0.01	0.4 ± 0.15	0.000*
ΔLaSr%	–0.05 ± 0.27	0.51 ± 0.18	0.000*
ΔLaScd%	0.09 ± 0.80	0.40 ± 0.27	0.044*
ΔLaSct%	–0.12 ± 1.08	0.74 ± 0.54	0.000*
Rest-HR	79.23 ± 12.96	71.23 ± 10.70	0.002*
Peak-HR	172.56 ± 15.04	173.40 ± 6.38	0.801

*p < 0.05. Rest-GLS, LV global longitudinal strain at rest stage; Peak-GLS, LV global longitudinal strain at peak stage; ∆GLS = Peak_GLS-Rest_GLS; ∆GLS% = (Peak_GLS-Rest_GLS)/Rest_GLS; Rest-LASr, LA reservoir strain at rest stage; Peak-LASr, LA reservoir strain at peak stage; Rest-LAScd, LA conduit strain at rest stage; Peak-LAScd, LA conduit strain at peak stage; Rest-LASct, LA contraction strain at rest stage; Peak-LASct, LA contraction strain at peak stage; ∆LASr% = (Peak_LASr-Rest_LASr)/Rest_LASr; ∆LAScd% = (Peak_LAScd-Rest_LAScd)/Rest_LAScd; ∆LASct% = (Peak_LASct-Rest_LASct)/Rest_LASct; Rest-HR, heart rate at rest stage; Peak-HR, heart rate at peak stage; HCM, hypertrophic cardiomyopathy.

### 3.3 Comparison of LA and LV Strain and Reserve Capacity among Three Group 

In the HCM-1 and HCM-2 groups, there was no statistically significant difference between the rest and peak. While, the LA and LV strain of the normal group was significantly different between the rest and peak (*p* < 0.05), the LA and LV strain had obvious reserve capacity (as shown in Table 4).

**Table 4.** **Comparison of left atrium and left ventricle strain and reserve capacity among three groups at rest and exercise**.

	HCM-1 (N = 36)			HCM-2 (N = 34)			Normal (N = 30)		
	REST	PEAK	*p* (1)	REST	PEAK	*p* (2)	REST	PEAK	*p* (3)
LASr	18.73 ± 5.76	17.59 ± 6.99	0.285	25.05 ± 5.23	23.13 ± 6.36	0.033	44.39 ± 8.20	66.61 ± 10.90	0.000*
LAScd	–10.69 ± 5.15	–10.12 ± 1.59	0.735	–17.11 ± 1.41	–15.58 ± 1.16	0.141	–27.97 ± 6.45	–38.32 ± 7.47	0.000*
LASct	–6.72 ± 1.07	–5.39 ± 0.77	0.313	–8.76 ± 0.91	–7.54 ± 1.02	0.174	–16.41 ± 3.73	–28.28 ± 9.20	0.000*
GLS	–19.59 ± 3.14	–17.16 ± 1.20	0.050	–20.58 ± 2.69	–20.75 ± 2.69	0.821	–25.25 ± 2.27	–35.67 ± 2.50	0.000*
SBP	124.0 ± 20.47	163.44 ± 30.09	0.000*	124.94 ± 18.98	175.18 ± 31.41	0.000*	121.87 ± 7.71	167.47 ± 14.22	0.000*
DBP	73 ± 14.97	74 ± 15.62	0.669	76.44 ± 13.01	73.65 ± 24.03	0.434	76.83 ± 7.15	77.10 ± 10.28	0.000*

*p < 0.05. GLS, LV global longitudinal strain; LASr, LA reservoir strain; LAScd, LA conduit strain; LASct, LA contraction strain; SBP, systolic blood pressure; DBP, diastolic blood pressure; HCM, hypertrophic cardiomyopathy.

The comparison among the three groups found that: the Rest-LASr, Rest-LAScd, Peak-LASr, Peak-LAScd, Peak-GLS, ΔLASct%, all satisfied: HCM-1 < HCM-2 < normal group (*p <* 0.05). Rest-LASct, Peak-LASct, Rest-GLS, ΔLASr%, LAScd% had no significant difference between HCM-2 group and HCM-1 group (as shown in Table 5). The Table 6 showed that: LASr and GLS showed high repeatability both intra- and interobserver variability.

**Table 5.** **Comparison of strain and reserve at the same state in HCM-1, HCM-2, normal group**.

	HCM-1 (N = 36)	HCM-2 (N = 34)	Normal (N = 30)	*p* (1-2)	*p* (1-N)	*p* (2-N)
Rest-LASr	18.73 ± 5.76	25.05 ± 5.23	44.4 ± 8.20	0.000*	0.000*	0.000*
Rest-LAScd	–10.69 ± 5.15	–17.11 ± 8.27	–28 ± 6.45	0.000*	0.000*	0.000*
Rest-LASct	–6.72 ± 1.07	–8.76 ± 0.91	–16.4 ± 3.73	0.155	0.000*	0.000*
Peak-LASr	17.59 ± 6.99	23.13 ± 6.36	66.61 ± 10.90	0.001*	0.000*	0.000*
Peak-LAScd	–10.12 ± 1.59	–15.58 ± 1.16	–38.3 ± 7.47	0.008*	0.000*	0.000*
Peak-LASct	–5.39 ± 0.77	–7.54 ± 1.02	–28.3 ± 9.20	0.097	0.000*	0.000*
Rest-GLS	–19.59 ± 3.14	–20.58 ± 2.69	–25.3 ± 2.27	0.163	0.000*	0.000*
Peak-GLS	–17.16 ± 1.20	–20.75 ± 0.67	–35.7 ± 2.50	0.013*	0.000*	0.000*
ΔLASr%	–0.03 ± 0.32	–0.07 ± 0.21	0.51 ± 0.18	0.640	0.000*	0.000*
ΔLAScd%	0.22 ± 1.04	–0.04 ± 0.40	0.40 ± 0.27	0.170	0.327	0.000*
ΔLASct%	–0.39 ± 0.90	0.17 ± 1.18	0.74 ± 0.54	0.028*	0.000*	0.014*
ΔGLS%	–0.12 ± 0.37	0.02 ± 0.24	0.42 ± 0.15	0.044*	0.000*	0.000*

*p < 0.05. Rest-GLS, LV global longitudinal strain at rest stage; Peak-GLS, LV global longitudinal strain at peak stage; ∆GLS = Peak_GLS-Rest_GLS; ∆GLS% = (Peak_GLS-Rest_GLS)/Rest_GLS; Rest-LASr, LA reservoir strain at rest stage; Peak-LASr, LA reservoir strain at peak stage; Rest-LAScd, LA conduit strain at rest stage; Peak-LAScd, LA conduit strain at peak stage; Rest-LASct, LA contraction strain at rest stage; Peak-LASct, LA contraction strain at peak stage; ∆LASr% = (Peak_LASr-Rest_LASr)/Rest_LASr; ∆LAScd% = (Peak_LAScd-Rest_LAScd)/Rest_LAScd; ∆LASct% = (Peak_LASct-Rest_LASct)/Rest_LASct; HCM, hypertrophic cardiomyopathy.

**Table 6.** **Intraobserver and Interobserver variability**.

	Intraobserver variability			Interobserver variability		
	ICC	95% Lower	95% Upper	ICC	95% Lower	95% Upper
Rest-LASr	0.982	0.973	0.988	0.975	0.963	0.983
Rest-LAScd	0.936	0.906	0.956	0.923	0.888	0.948
Rest-LASct	0.713	0.601	0.797	0.684	0.564	0.776
Peak-LASr	0.948	0.924	0.965	0.977	0.966	0.985
Peak-LAScd	0.974	0.962	0.983	0.809	0.729	0.867
Peak-LASct	0.840	0.772	0.89	0.723	0.614	0.805
Rest-GLS	0.857	0.795	0.901	0.826	0.752	0.879
Peak-GLS	0.925	0.891	0.949	0.933	0.902	0.954

Rest-GLS, LV global longitudinal strain at rest stage; Peak-GLS, LV global longitudinal strain at peak stage; Rest-LASr, LA reservoir strain at rest stage; Peak-LASr, LA reservoir strain at peak stage; Rest-LAScd, LA conduit strain at rest stage; Peak-LAScd, LA conduit strain at peak stage; Rest-LASct, LA contraction strain at rest stage; Peak-LASct, LA contraction strain at peak stage; ICC, intraclass correlation coefficient.

### 3.4 Comparison of METS among the Three Groups

**Fig. 2.**
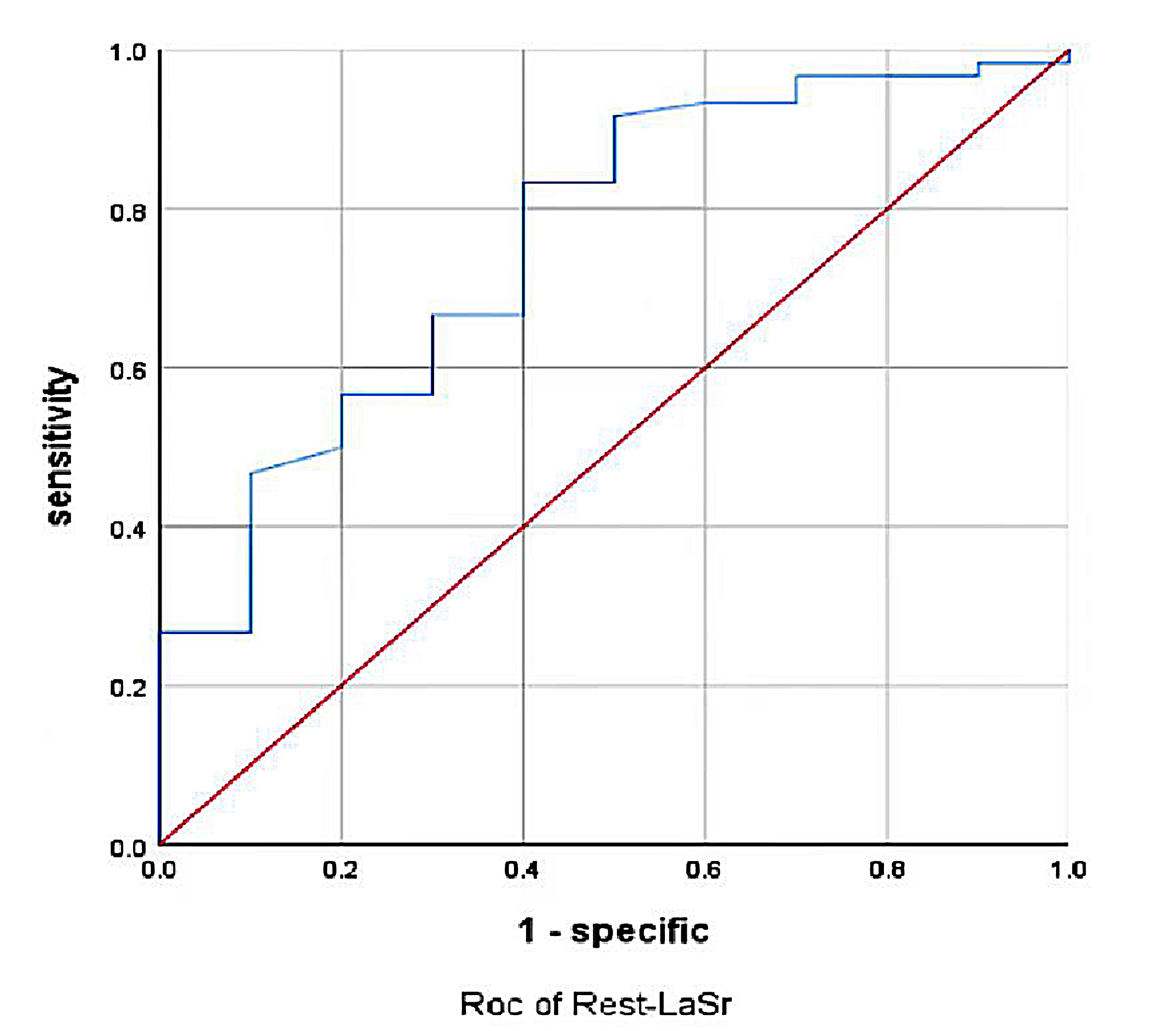
**Rest-LASr predicted METS less than 6.0 ROC curve**. LASr, LA reservoir strain; METS, metabolic equivalents.

**Fig. 3.**
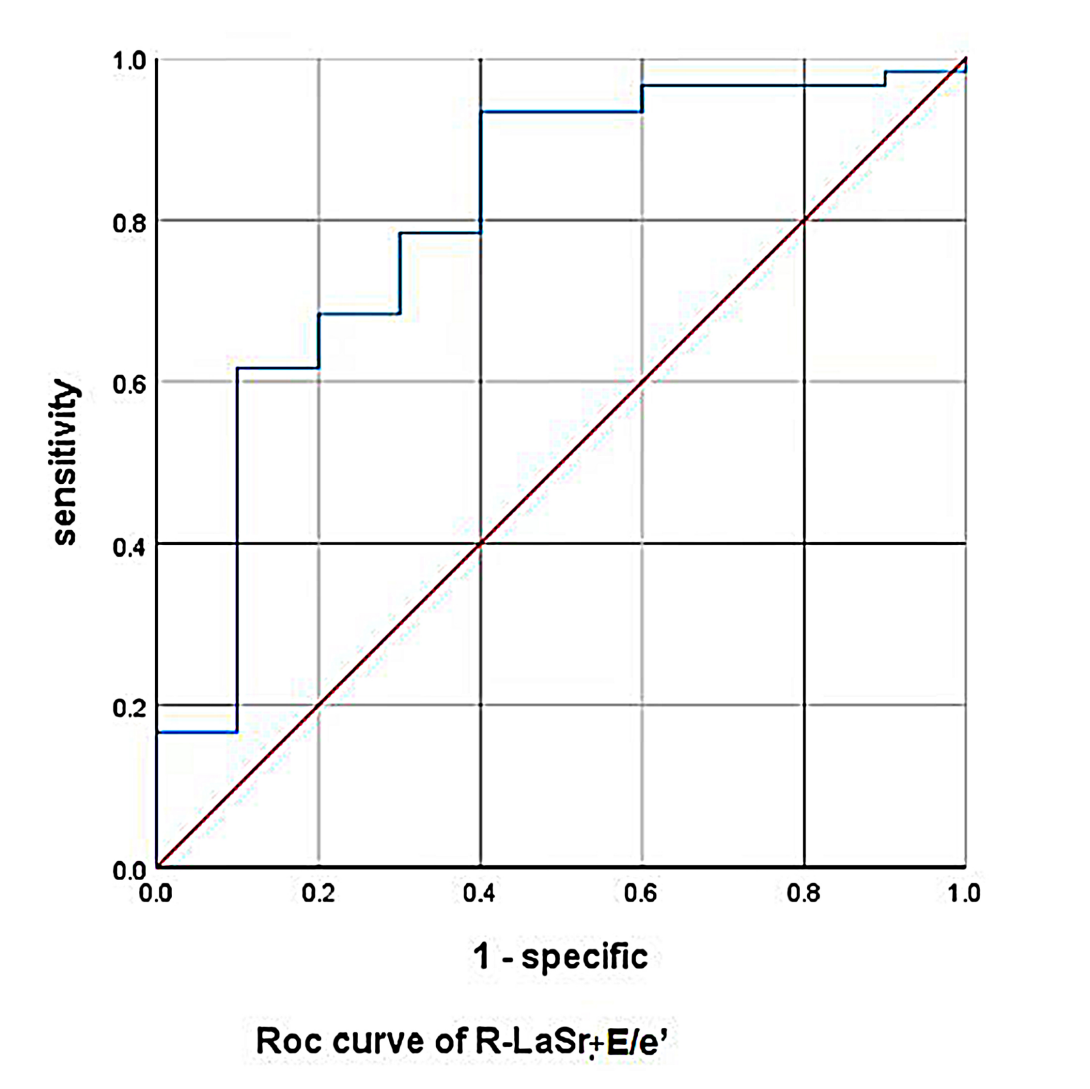
**Rest-LASr+E/e’-rest predicted METS less than 6.0 ROC curve**. LASr, LA reservoir strain; METS, metabolic equivalents.

In this study, 9 patients (25%) in the HCM-1 group had a METS <6.0 and 27 patients (75%) had a METS >6.0. In contrast, in the HCM-2 group, there was one patient (2.94%) with a METS <6.0 and 33 patients (97.06%) with a METS >6.0. The mean METS score in the HCM-1 group was significantly smaller than that in the HCM-2 group (7.91 ± 2.76 vs 10.25 ± 2.00, *p <* 0.05; Table 2); there was no significant difference between the HCM-2 group and the normal group (10.25 ± 2.00 vs 10.46 ± 0.05, *p >* 0.05). The Rest-LASr indicated the highest differential diagnostic performance for METS <6.0 (area under curve [AUC]: 0.759); the AUC of the composite model Rest-LASr+E/e’-rest was 0.8 (as shown in Figs. 2,3 and Tables 7,8).

**Table 7.** **Sensitivity and specificity analysis**.

	Sensitivity	Specificity	Cutoff	AUC	95% Lower	95% Upper
Rest-LASr	0.833	0.6	16.91	0.759	0.599	0.919
E/e’-rest	0.783	0.7	17.06	0.757	0.572	0.941
Rest-LAScd	0.567	0.7	–12.89	0.648	0.46	0.836
Rest-LASct	0.417	0.9	–10.24	0.593	0.418	0.767
Rest-GLS	0.517	0.8	–20.41	0.671	0.501	0.841

Rest-GLS, LV global longitudinal strain at rest stage; Rest-LASr, LA reservoir strain at rest stage; Rest-LAScd, LA conduit strain at rest stage; Rest-LASct, LA contraction strain at rest stage; E-rest, Early diastolic forward mitral flow velocity at rest stage; e’-rest, early-diastolic mitral annular velocity (e’ was calculated as the mean of the septal e’wave and lateral e’ wave by using pulsed wave-tissue Doppler imaging) at rest stage.

**Table 8.** **Multivariate joint sensitivity and specificity analysis**.

	Sensitivity	Specificity	AUC	95% Lower	95% Upper
Rest-LASr+Rest-LASct	0.933	0.5	0.755	0.587	0.923
Rest-LASr+Rest-LASct+Rest-LAScd	0.933	0.5	0.753	0.585	0.922
Rest-LASr+E/e’-rest	0.933	0.6	0.8	0.638	0.962
Rest-LASct+E/e’-rest	0.833	0.8	0.795	0.612	0.978
Rest-LASr+Rest-LASct+Rest-LAScd+E/e’-rest	0.917	0.7	0.797	0.617	0.976

Rest-LASr, LA reservoir strain at rest stage; Rest-LAScd, LA conduit strain at rest stage; Rest-LASct, LA contraction strain at rest stage; E-rest, early diastolic forward mitral flow velocity at rest stage; e’-rest, early-diastolic mitral annular velocity (e’ was calculated as the mean of the septal e’wave and lateral e’ wave by using pulsed wave-tissue Doppler imaging) at rest stage.

### 3.5 Correlation Analysis

**Fig. 4.**
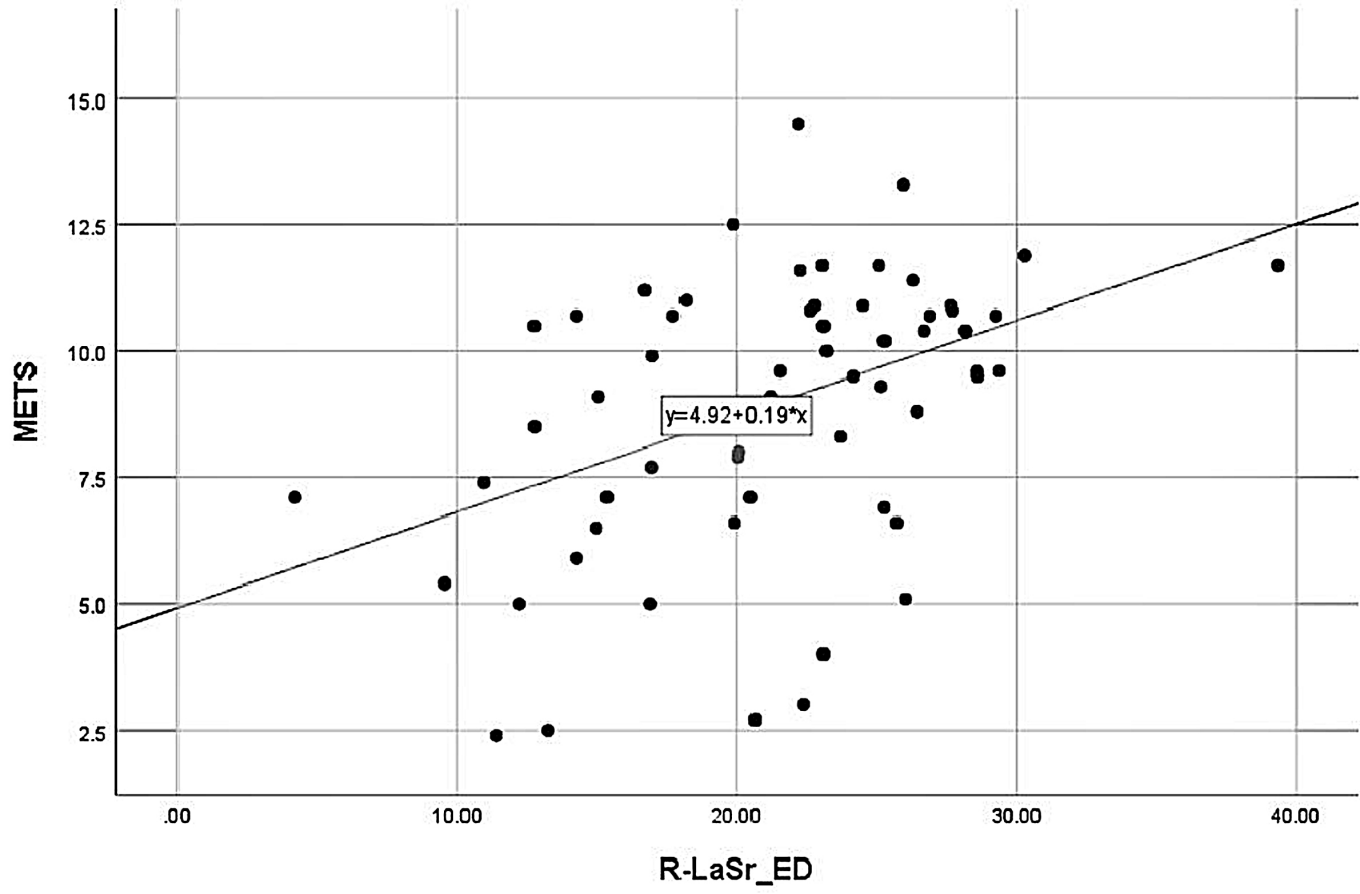
**METS correlation with Rest-LASr**. METS was positively correlated with Rest-LASr. LASr, LA reservoir strain; METS, metabolic equivalents.

**Fig. 5.**
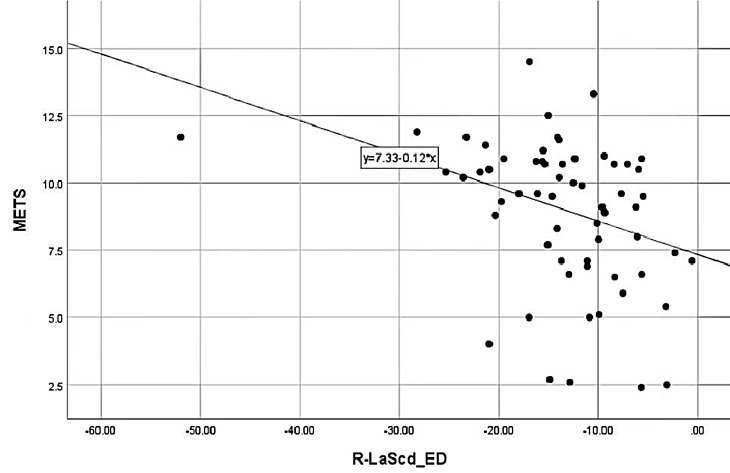
**METS correlation with Rest-LAScd**. METS was negatively correlated with Rest-LAScd. LASr, LA reservoir strain; METS, metabolic equivalents.

METS was positively correlated with Rest-LASr (r = 0.448), Peak-LASr (r = 0.538), ΔLASr% (r = 0.325), ΔLaSct% (r = –0.268); METS was negatively correlated with age (r = –0.494), E/e’-rest (r = –0.450), Rest-LAScd (r = –0.392), Peak-LAScd (r = –0.371), Peak-LaSct% (r = –0.291), GLS (r = –0.338), BMI (r = –0.312) (as shown in Figs. 4,5).

## 4. Discussion

LV diastolic dysfunction includes insufficient or brady relaxation in the early stages of disease progression, and in advanced stages of disease, manifests mainly as reduced compliance and increased stiffness. These changes led to increased LV filling pressure and subsequently, may cause an increase in LA and pulmonary venous pressure. LA reservoir strain occurs during systole when the pulmonary veins fill the LA, thus causing the LA wall to stretch; this corresponds to LV isovolumic contraction and isovolumic relaxation. LA conduit strain occurs in the early diastolic period; when the mitral valve is opened, the LA blood content flows into the LV; this refers to the rapid filling period and the slow filling period which is regulated by LA compliance (LV relaxation and compliance). LA contraction strain is dependent on venous return and LV end-diastolic pressure [14, 15]. LAS alterations tend to progress between all stages of diastolic dysfunction. In particular, reservoir strain is significantly better than GLS and E/e’ and has been shown to predict cardiovascular events and represent a more accurate diagnostic tool to help classify diastolic dysfunction [16, 17, 18, 19, 20, 21, 22].

**Fig. 6.**
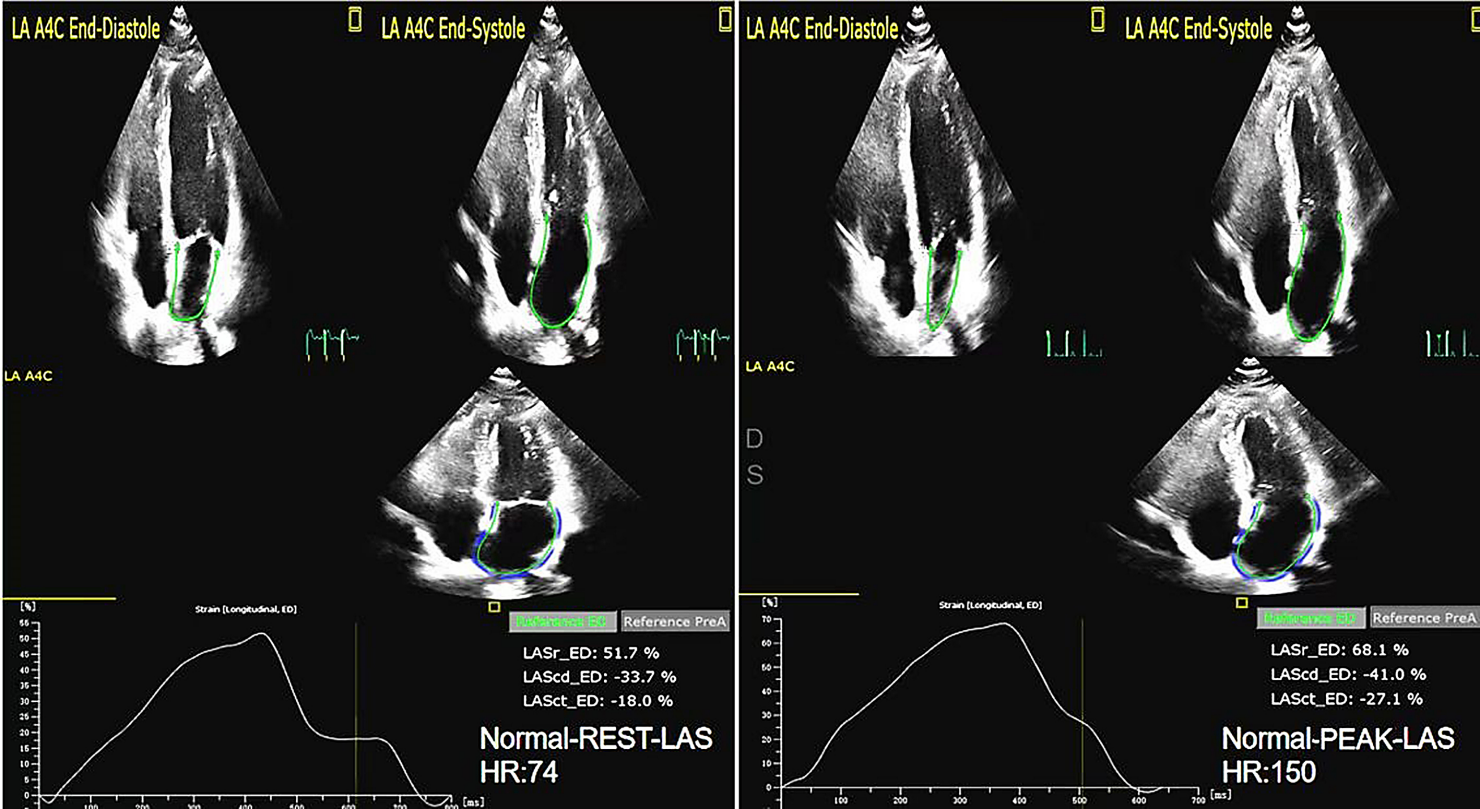
**The LAS of rest and peak in Normal group**. LAS, left atrial strain.

**Fig. 7.**
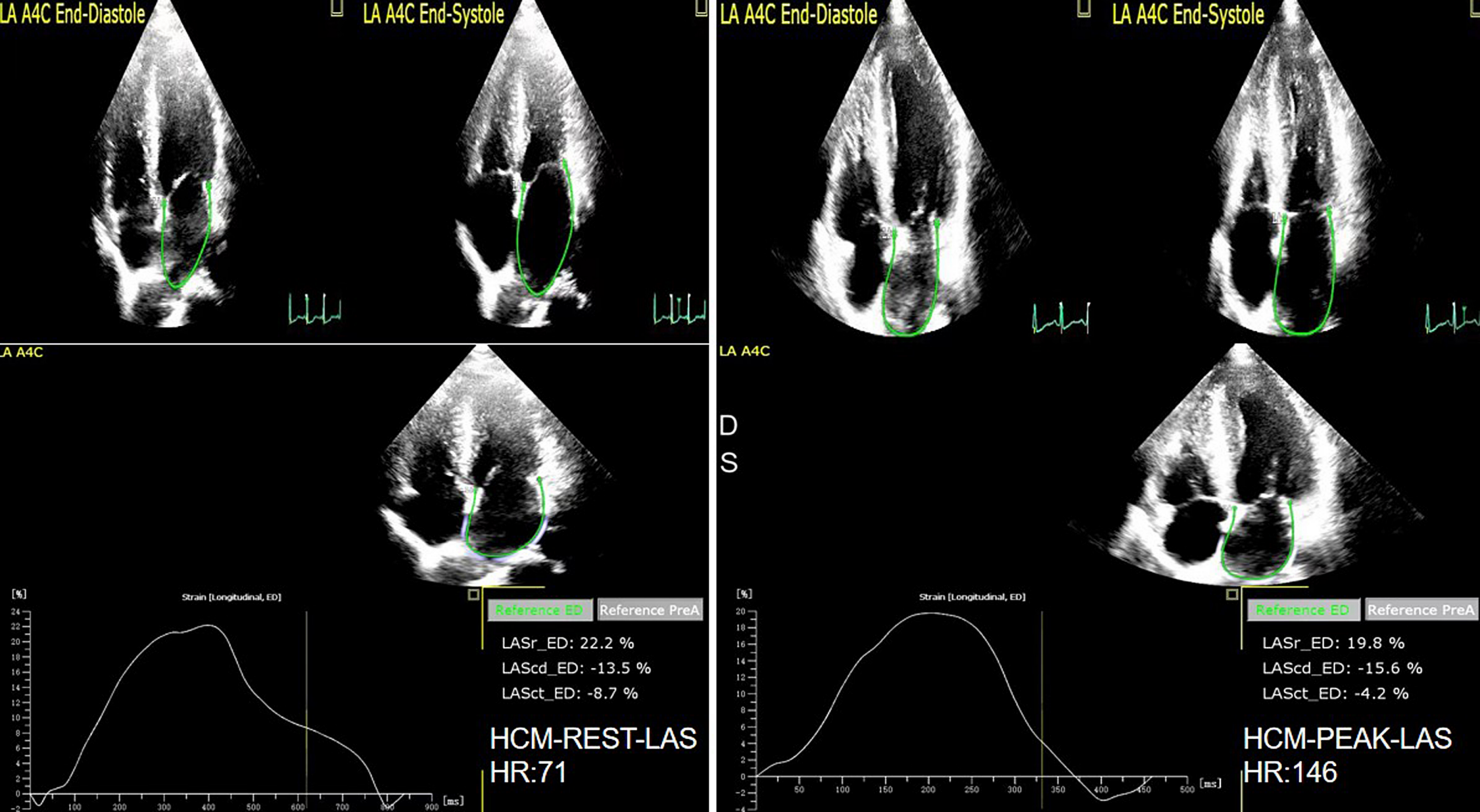
** The LAS of rest and peak in HCM group**. LAS, left atrial strain.

A previous systematic analysis and meta-evaluation of 2452 healthy subjects [23] showed that the normal reference range for LA reservoir strain was 39% while that of conduit strain was 23% and contraction strain was 17%. Our current analysis yielded similar outcomes: the LA reservoir, conduit and contraction strain were 44.39 ± 8.20%, –27.97 ± 6.45%, and –16.41 ± 3.73%, respectively. A previous study [24] showed that when the LA reservoir strain was <19%, there was a significant association between increased LV filling pressure and diastolic dysfunction. Another study [25] showed that a LA reservoir strain <18% and contraction strain <8% were more predictive of increased LV filling pressure than LAVI and conventional doppler parameters (*p* < 0.05). Furthermore, the accuracy of diagnosed elevated LV filling pressure with only reservoir strain was 75%, the accuracy of diagnosed elevated LV filling pressure with only contraction strain was 72%, and the accuracy of LAS combined with conventional parameters in diagnosed elevated LV filling pressure was 82%. Gillebert *et al*. [26] reported that reduced LA reservoir function was a sensitive marker of early diastolic dysfunction and the coexistence of low reservoir and low contraction function indicated more severe heart failure, atrial fibrillation, thrombotic complications, acute heart failure syndrome and even death. When LV diastolic function decreases, end-diastolic volume increases, thus leading to a reduction in LA reservoir strain. Therefore, a reduction in LA reservoir strain may reflect a reduction in LV diastolic function at a time point much earlier. In the HCM-2 group, although LA and LAVI were still within the normal range, the resting E/e’ was also between 8 and 14; furthermore, the resting LA reservoir strain (25.05 ± 5.23% vs 44.39 ± 8.20%), conduit strain (–17.11 ± 8.27% vs –28 ± 6.45%) and contraction strain (–8.76 ± 0.91% vs –16.4 ± 3.73%) were all significantly lower than normal group (*p* < 0.001), thus indicating that the reservoir, conduit, and contraction function were reduced (as shown in Figs. 6,7). The reduction of LA reservoir strain indicated that LA relaxation and compliance had been reduced, therefore suggesting that patients in the HCM-2 group may have entered the early stage of LV diastolic hypofunction. In this study, the LA conduit and contraction strain in the HCM-2 group were reduced, thus indicating that the compliance of the left atrium was reduced. The LA can reduce the function of the conduit by increasing the end-diastolic contraction compensation. In late diastole, the LA myocardial contraction was reduced, the ability to actively pump blood to the LV was reduced, and the contraction function of the LA was also reduced. We also found that the HCM-2 group had “lighter” impairment of the LA reservoir and conduit strain than the HCM-1 group; importantly, there was no difference between the HCM-1 and HCM-2 groups with regards to LA contraction strain (*p* > 0.05). Thus, it is possible that LA contraction strain was not sensitive enough for the detection of reduced diastolic function. LA contraction function is a biphasic process in the course of the disease; when diastolic function is damaged in the early stage, the LA contraction function still has a certain reserve which can increase the contraction capacity. As the diastolic function further decreased, the LA pressure further increased, exceeding the reserve capacity of LA contraction; thus, LA contraction dysfunction and LA contraction strain decreased. Therefore, when the E/e’, as measured by conventional echocardiography, is between 8 and 14, if the LAS is measured in combination, the diastolic function can be quickly and accurately assessed, thus meeting the needs of clinical work for predicting cardiac function.

Patel *et al*. [27] showed that the lack of LA functional reserve and reduced LV diastolic function were associated with reduced exercise capacity. In the present study, we found that there was no statistical difference in the LA reservoir strain, conduit strain, and GLS in the HCM-1 group when compared between peak level and the rest stage. Moreover, there was no statistical difference in LA reservoir strain or conduit strain in the HCM-2 group when compared between peak level and the rest stage, thus indicating that the reserve capacity of the LA reservoir, conduit, contraction strain and GLS were reduced in both groups. It was also found that at rest and peak stages, the LA reservoir strain and conduit strain in the HCM-2 group was higher than in the HCM-1 group. In the HCM-2 and HCM-1 groups, the LA contraction strain reserve (ΔLASct%) and the ΔGLS% were significantly lower than in the normal group; furthermore, the ΔLASct% and ΔGLS% of the HCM-2 group were higher than that the HCM-1 group (*p* < 0.05). The Rest-LASr, Rest-LAScd, Peak-LASr, and ΔLaSct% were all positively correlated with METS; these indices were significantly higher in the HCM-2 group than in the HCM-1 group (*p* < 0.05). These findings are consistent with the fact that the METS in the HCM-2 group was higher than that in the HCM-1 group and that there was no statistical difference in METS when compared between the HCM-2 group and normal group. The better METS in the HCM-2 group can be attributed to the higher reservoir strain and conduit strain both in the rest and peak stages, and the Peak-GLS in the HCM-2 group in addition to the higher contraction strain reserve capacity and the higher ΔGLS%. The contraction of skeletal muscle after exercise promoted a greater amount of venous return to the right atrium. The contraction strain reserve of the HCM-2 group was still able to compensate for the left ventricle by increasing the atrial contraction and pumping blood to make up for the stroke volume with reduced diastolic function. Furthermore, there was a greater Peak-GLS in the HCM-2 group after exercise, thus indicating enhanced left ventricular contraction function. Although the METS in the HCM-2 group and normal group were not significantly different, the real hemodynamics and pathophysiological mechanisms underlying the METS were different; thus, the risk of cardiovascular events also differed.

In a previous study, Badran *et al*. [28] found that in a normal group, the rest and peak HR were 82 ± 8 and 165 ± 1, the rest and peak SBP were 118 ± 3 and 155 ± 7, and the rest and peak DBP were 79 ± 4 and 97 ± 4l. However, in the HCM group, the rest and peak HR were 70 ± 11 and 142 ± 21, the rest and peak SBP were 128 ± 22 and 145 ± 32, and the rest and peak DBP were 81 ± 14 and 81 ± 12. Analysis showed that both the rest and peak SBP differed significantly between the normal and HCM groups. In the present study, we obtained similar results; in the normal group, the rest and peak HR were 71 ± 10 and 173 ± 6, the rest and peak SBP were 120 ± 8 and 168 ± 15, and the rest and peak DBP were 78 ± 8 and 77 ± 11. However, in the HCM group, the rest and peak HR were 79 ± 11 and 172 ± 15, the rest and peak SBP were 126 ± 24 and 172 ± 27, and the rest and peak DBP were 77 ± 12 and 75 ± 17. Only rest SBP differed significantly between the normal and HCM groups (Table 1). For the HCM-1, HCM-2, and normal groups, we observed significant differences for SBP before and after exercise (Table 4). During exercise, the sympathetic nerve is excited, the HR increased, the heart pumps more blood, the pressure of blood vessel wall increases, which can lead to increased blood pressure. The peak SBP does not exceed 190–210 mmHg at normal, while the DBP basically does not change or slightly decreases. Because SBP is the pressure of blood against the walls of blood vessels when the heart contracts. During exercise, the circulation speeds up and the volume of heart strokes increases. The more blood the heart pumps out, the more pressure is created against the walls of blood vessels, so the SBP rises. DBP mainly reflects vascular elasticity and peripheral circulation resistance. In order to obtain more oxygen during exercise, the body reduces the resistance generated by peripheral arterioles and increases the blood supply to the exercise system through blood pressure regulation mechanism, thus making DBP stable or slightly decreased.

**Fig. 8.**
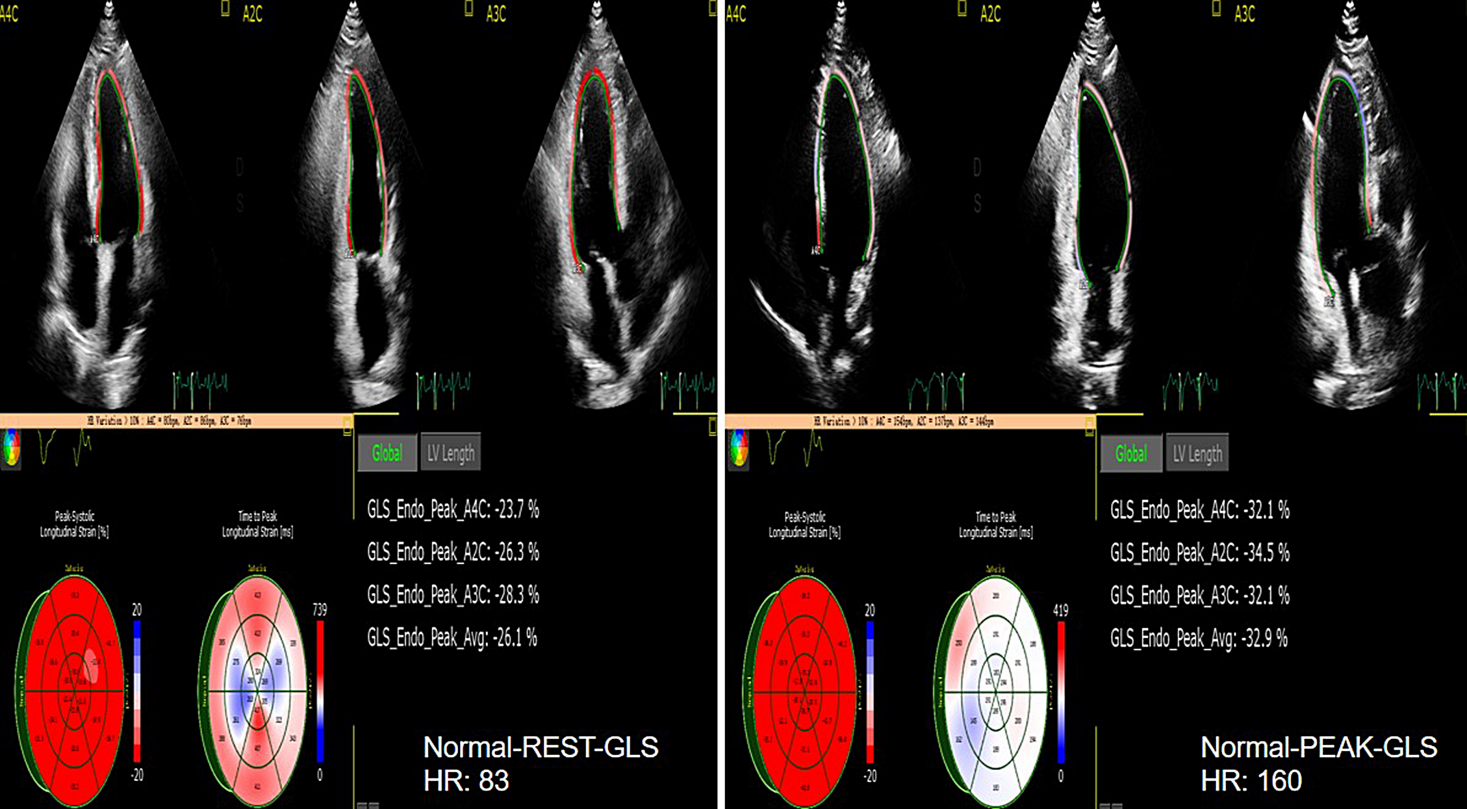
**The GLS of rest and peak in normal group**. GLS, LV global longitudinal strain.

**Fig. 9.**
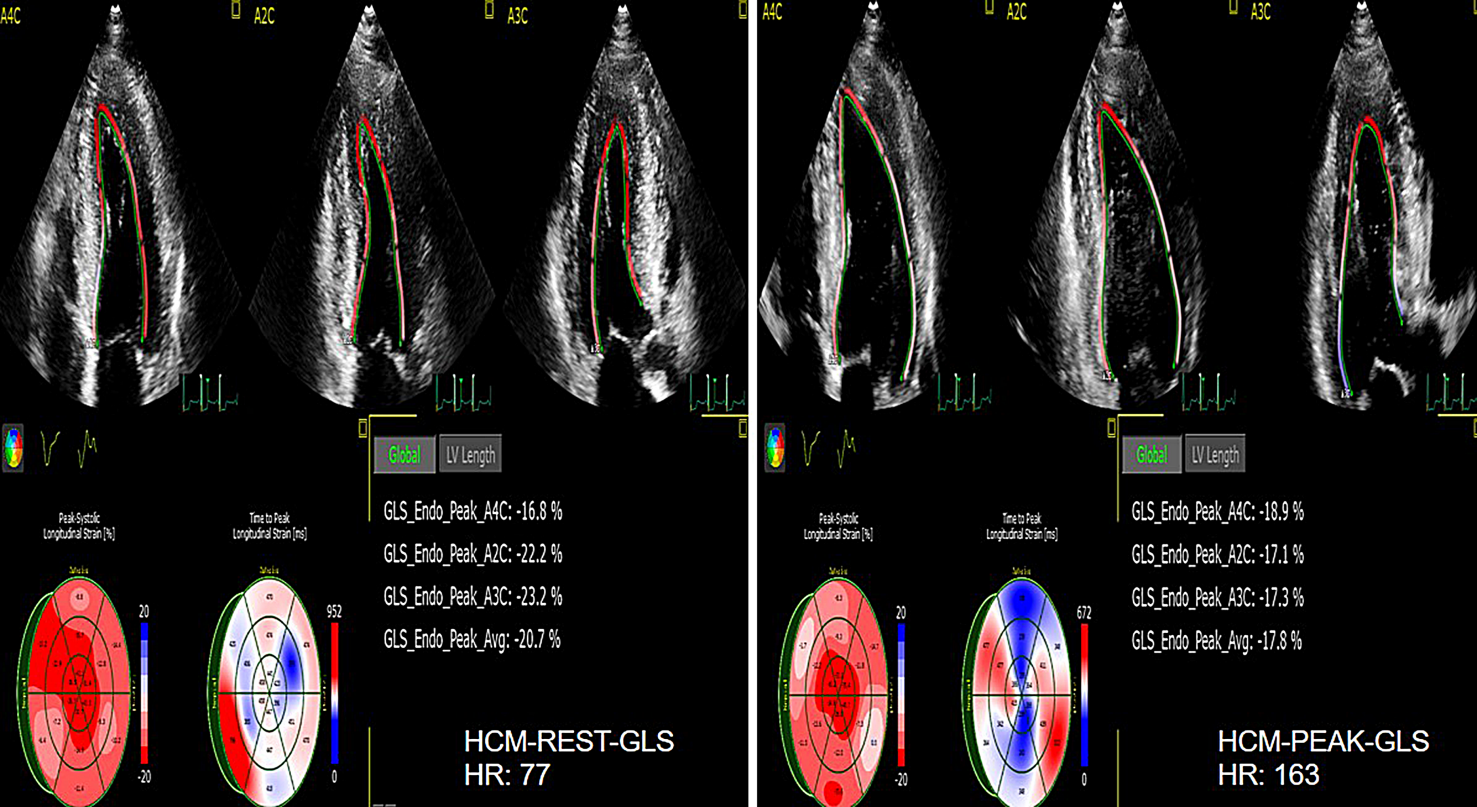
**The GLS of rest and peak in HCM group**. GLS, LV global longitudinal strain; HCM, hypertrophic cardiomyopathy.

We found that the EDV in the HCM group at rest and peak stages was higher than in the normal group. Furthermore, the LV-GLS and LAS in the HCM group at the peak stage were smaller than in the rest stage. Thus, from a mechanical point of view, the peak myocardial contractility was reduced, therefore the EDV increased. In a previous study, Wu *et al*. [29] study found that in a normal group, the rest and peak LV-GLS were –20.8 ± 2.1 and –27.6 ± 1.9, ΔGLS was –4.75 ± 1.78, while in the HCM group, the rest and peak LV-GLS were –17.1 ± 2.8 and –20.0 ± 3.3, ΔGLS was –2.93 ± 1.58, the age of the subject was 52 ± 12, and the LV-GLS increased after exercise in both the normal and HCM groups. Badran *et al*. [28] found that in a normal group, the rest and peak LV-GLS were –18.5 ± 2 and –23.1 ± 2.7, ΔGLS was –3.4 ± 1.13, furthermore, in the HCM group, the rest and peak LV-GLS were –13.5 ± 5.8 and –11.8 ± 4.9, ΔGLS was –3.23 ± 5.0, the age of the subjects was 42 ± 12, and the LV-GLS increased after exercise in the normal group but decreased after exercise in the HCM group. Mahfouz *et al*. [30] found that in a normal group, the LA reservoir, conduit, and contraction strain were 52.1 ± 8.2%, –27.5 ± 4.4%, and –21.5 ± 4.0%, respectively. Similar results were obtained in the present study: in the normal group, the rest and peak LV-GLS were –25.25 ± 2.27 and –35.67 ± 2.5, while in the HCM group, the rest and peak LV-GLS were –20.07 ± 2.95 and –18.91 ± 6.09, respectively. The LV-GLS increased after exercise in the normal group but decreased after exercise in the HCM group (as shown in Figs. 8,9). LA reservoir, conduit and contraction strain were 44.39 ± 8.20%, –27.97 ± 6.45%, and –16.41 ± 3.73%, respectively. A previous study found that ΔGLS = GLS_peak-GLS_rest, but we consider that the percentage of the increase is more reasonable and accurate; therefore, in our study, we used the ΔGLS%, ΔLASr%, ΔLAScd%, ΔLASct%; these values were 40%, 51%, 40%, and 74%, respectively. Although it seems that the LV-GLS data from the normal group in our study was larger than that in the two previous studies, it is also important to consider that LV-GLS may be affected by age, race, gender; furthermore, the sample size of our normal group was 30 and may have had an impact on our findings. The blood flow of the left atrium increased sharply in the supine position immediately after the exercise stopped, and the deformation of the left atrium reached its maximum state, the following factors were taken into consideration, which would also lead to increased the LA strain after exercise cessation: (1) with the increase of intensity during exercise, the contractile force of the heart is enhanced, and the venous return to right atrium is increased; (2) the contraction and diastole of skeletal muscle during exercise can lead to the increase of venous return, (3) with the increase of intensity of exercise, the intrapleural pressure is negative pressure, the transmural pressure of the large vein in the thoracic cavity is larger, during inspiration, the thoracic volume is increased, and the negative pressure value of the pleural cavity is further increased. The large vein in the chest cavity and the right atrium are more dilated, the pressure is further reduced, the blood flow in the peripheral vein returns to the right atrium, and the blood flow from the pulmonary vein into the left atrium increases, (4) after the peak stage, the patient immediately shifts to the left decubitus position, the venous return flow increases. Genovese *et al*. [2] found that: in the normal group, LA strain is load dependent but to a lesser degree than LA volume, LA strain had relative advantage over LV volume in diagnostic paradigm. The dynamic images we collected were clear and were carried out in strict accordance with the analytical process; when heart rate reached approximately 170 bpm at the peak stage of exercise, we analyzed the images three times and took the mean value. All data are true and effective. At present, there are very few studies on left atrial strain after exercise. Therefore, we need to further expand the sample size, acquire the reference range of left atrial strain in healthy subjects after exercise, and facilitate the improvement of our follow-up research.

**Fig. 10.**
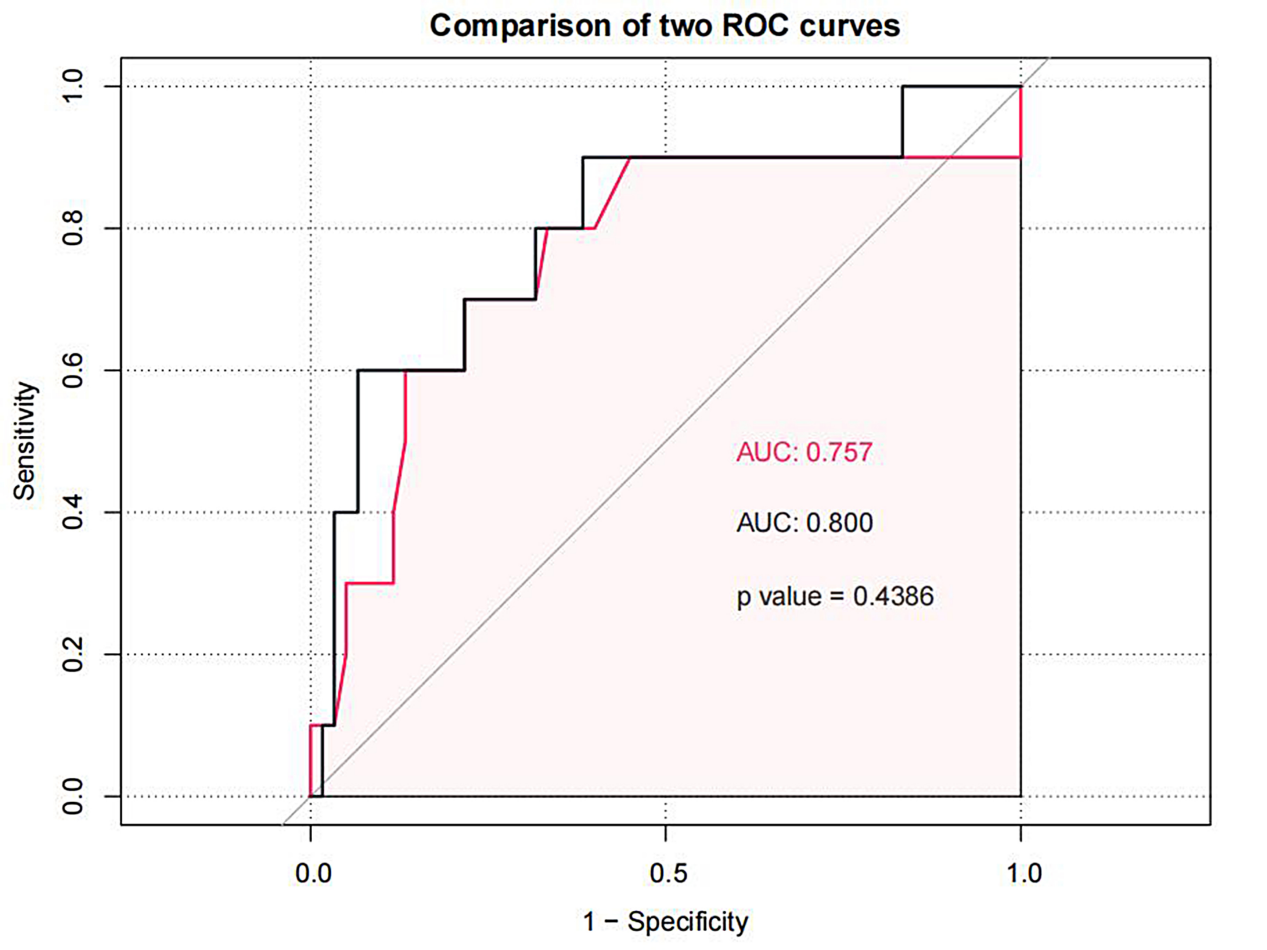
**Comparison the AUC of E/e’ and LASr+E/e’**. LASr, LA reservoir strain; E/e’, the ratio of early diastolic forward mitral flow velocity and early-diastolic mitral annular velocity.

As shown in Figs. 2,3 and Tables 6,7, the AUC for LaSr was 0.759 (sensitivity 0.833, specificity 0.6, cutoff value –16.91%) and had the greatest discriminatory value. However, the specificity of LaSr was low, although the sensitivity and AUC were high enough to reduce the chances of missed diagnosis. In this study, when the AUC of E/e’ was 0.757, the cutoff value was 17.06, at this time, the index E/e’ and the AUC cannot be used in patients with E/e’ between 8 and 14. Therefore, we need to add LASr into the model to assist clinical judgment. When Rest-LASr+E/e’-rest was used as the prediction model, the AUC was 0.800. Although E/e’ and LASr+E/e’ had similar AUC values (0.757 vs 0.800, *p* = 0.4386) (as shown in Fig. 10) , the technology and principle for obtaining E/e’ and LASr are different. E/e’ was obtained through pulsed wave-tissue Doppler imaging, this technique is angle dependent. In addition, E/e’ can be affected by valve and left ventricular outflow tract obstruction, and was limited in terms of clinical application [23]. LA strain is based on speckle tracking technology which can quantitatively evaluate mechanical function of the atrial myocardium by analyzing myocardial deformation and can analyze the working characteristics of the atrial myocardium in different cardiovascular diseases; furthermore, this parameter has been shown to have predictive value for the risk of adverse cardiovascular events. LASr can be accurately measured even in combination with valve disease, heart failure and arrhythmia; these conditions are more common in clinical diagnosis and treatment. Furthermore, speckle tracking echocardiography has become an increasingly standard imaging method in routine clinical practice [31]. Moreover, LASr is independent of LAV, LV-GLS, age, LVEF, and E/e’ [32]. Thus, when E/e’ lies between 8 and 14, the routine assessment of left atrial function can be an important strategy to supplement the current prediction models.

## 5. Conclusions

In conclusion, analysis showed that when the E/e’ was between 8 and 14, the LAS and reserve capacity of HCM patients were significantly reduced. Our findings suggest that the routine assessment of LAS +E/e’ can be a strategy with which to supplement current predictive models and facilitate clinical management strategies.

## 6. Limitation

This study was limited to a single center sample, and many simultaneous factors affected METS, such as systemic inflammation, endothelial dysfunction, changes in intracellular and extracellular structures of cardiomyocytes, skeletal muscle bioenergetics, pulmonary functional status, mitral and tricuspid regurgitation, left ventricular outflow tract obstruction,etc, the pathophysiological changes of HCM may result in the above problems, which we did not refine analysis. At the same time, due to the different algorithms of the analysis software of different ultrasonic diagnostic instruments, the measurement results of LAS will be affected. It may be necessary to further expand the sample size and expand the ultrasonic diagnostic instrument model and analysis software to analyze LAS and METS in HCM patients, and follow-up to observe the occurrence of cardiovascular events in HCM patients. Another factor to consider is that the assessment of exercise tolerance was only based on METS. Other indices, such as the maximal oxygen consumption and the minute ventilation/carbon dioxide production slope, were not used. The original purpose of this study was to focus on the “grey uncertain population” with an E/e of 8–14 in order to quickly and accurately assist in the judgement of cardiac diastolic function and to predict exercise tolerance; thus, we considered METS as the short-term result. In clinical work, if it is necessary to predict the exercise tolerance but the patient cannot perform exercise tests (due to disability or other reasons), we can also consider using the LASr+E/e’ model to estimate the exercise tolerance in an approximate manner and provide a basis for clinical treatment decisions. Of course, we are also continuing to follow-up these patients and continue to observe long-term cardiovascular events for further detailed research reports.

## Data Availability

The data used to support the findings of this study are available from the author upon request.
